# Connecticut Valley Dental Society

**Published:** 1884-01

**Authors:** A. M. Ross


### CONNECTICUT VALLEY DENTAL SOCIETY.

(Continued from December Number.)

The evening session was devoted to a paper by Dr. J. L. Williams, of New Haven, Connecticut, upon "Embryology, with special reference to the development of teeth," illustrated by stereopticon. The paper was a fine presentation of the subject, being a full and complete statement of the most recent views, the result of careful investigation. The photographs, projected upon the screen were taken from Camera Lucida drawings.

*Dr. Atkinson*—I regard the presentment just made by Dr. Williams as the most concise and clear statement of the subject that I have ever been favored with, and I now render him my personal thanks.

Ever since the segmentation process of incubative evolution has been known, it has been regarded as a forestep in the production of tissues and their combination into organs and systems. Segmentation, as a forestep to organization, involves the necessity of other foresteps as cancellations of like predecessors, as chaotifications of the separate processes without losing the increment of the power, the limitations of which constitute the lay out or type of body to be formed according to the seriations in order, class, genera, species and variety, demanded by the germ under investigation. The germ is an impregnated or vivified egg. The egg is femenoid semen, and is vivified by admixture with another primate known as zoon, or spermatozoon—masculoid semen. The molecular metamorphosis through which egg and spermatazoon become capable of flowing together or mutually interpenetrating the other to constitute a germ capsule of evolution into a body like the parents from which it sprang, is too occult and involved to be entered upon with any hope of full apprehension and appreciation in the time and with the means at command. Let us say, however, that *ripening* is the term used by physiologists to indicate the concussion or churning together of the elements of the semens to awaken and engage definite measures of atomic energies, held in molecular grasp of bonds of affinity to constitute the feminoid and masculoid semens as conjointly and alternately quiescent reservoirs of plus and minus energy, ready to be awakened to form a germ upon being brought within the sphere of each other's influence.

Ovum and spermatazoon are definable and provable bodies with very small knowledge of natural history. But germs are difficult of identification in the quiescent condition, and infinitely varied in appearance during incubation or hatching. The three embryonal or germinal layers or sheets constitute important foresteps in incubative procession. Enfoldings and duplications of the epiblast or external sheet give the lay out of skin and body walls, or "somatopleure." Duplications of the hypoblast give the limit of the visceral walls, or "splanknopleure." The forward fold or invagination of the hypoblast forms the fore-gut, and the hind fold the hind-gut, and the œsophagus, stomach, duodenum, jejunum, ilium, colon and rectum are further differentiations of this layer.

I am rapidly getting over being offended by the mistakes made by earnest and honest naturalists. Naturalists as a class take the palm for dead earnestness to get at the truth in the fields they explore. Even a misinterpretation of any body or process may prove useful as a means of demanding review of the subject. Time would fail us were we to attempt to enumerate the so-called "demonstrations made before learned assemblages of the limits of possibility," that proved upon due examination to be but foresteps in negation of the veritable facts in science which prove the philosophy upon which SCIENCE is erected! When such great names as those of Rashkow, Goodsir,

Huxley and such ilk, fall into misinterpretations of embryology, may we not forgive Magitot, Dean and ourselves for palpable blunders? Palpable to those better endowed and better equipped for the investigations than ourselves.

The interior of the mouth and the anus are the points of punctures of the foldings of the epiblast and the hypoblast, between which the mesoblast or source of somites takes its rise; the division of mouth and pharynx and of the anal sphincters are the points where the epi and hypoblasts flow into each other ; the epiblast providing the lining of the mouth and the orifice of the anus, and the hypoblast supplying the embryonic corpuscles from which the mucous membrane of the pharynx and the termination of the rectum inside of the sphincters is formed. Embryology has not been sufficiently elaborated by patient and voluminous study to clear up all the appearances that mark the production of provisional and permanent tissues of the body. The great step in advance in embryological changes is the discovery by Heitzmann, of the tissual character of protoplasm, as opposed to the old interpretation of homogeneity of character. He says: "Protoplasm is a tissue made up of granules and strings, and a non-living watery fluid." I agree that protoplasm is an organized tissue, but not of non-living and living elements. I regard the fluid portion as of molecular constitution, and therefore a veritable living mass, whether we can demonstrate its structure to sight or not. The granules and strings are but the functions of the walls of the little sacs which hold the more fluid or watery part of the mass of protoplasm. Amœba, and all protoplasmic masses show this to sight by proper management. In microscopical examination of very small bodies, oblique light sometimes reveals structure that is invisible or obscure in the direst ray. Where objects are difficult to resolve we may gain much help in interpretation of their intimate structure by taking analogous specimens, the character of which is known to us. This is my justification for advising the use of soap-suds on the slide, to simulate or personate, for the purpose, an amoeba or mass of protoplasm. We know that air is the interior of the bubbles, and a thin structure of suds constitutes the periphery or sac which holds the air (an elastic gaseous body) in constraint. The similarity of appearance of this known structure to protoplasm, indicates the sacular character in the mass under dispute. The mind, imbued with a dominating desire to know the whole truth, whether it agrees with preconceptions or not, is in better state to get it than those who decide before what "ought to be."

Adjourned to half-past nine A. M. Thursday.

MORNING SESSION—SECOND DAY.

The Society met at half-past nine o'clock, Section Three being called. A paper entitled "Aconite" was read by Dr. George A. Maxfield, of Holyoke. It was voted at its conclusion to defer discussion, and Section Four was called, when a paper entitled "Tooth Crowning" was read by Dr. E. Parmly Brown.

*Dr. L. D. Shepard* was impressed by the paper charts and models exhibited, that there was something good in this thin platinum band. It is a nice thing to conform closely to the roots. He had had some experience in crown-setting, and the only criticism he could offer upon Dr. Brown's method would be that a large assortment of crowns would have to be made and kept on hand as a stock, to select the suitable crown and band from for each individual case. A nicely fitting band is the first essential, and is in reality the part of the work where the greatest accuracy is necessary. Failures of this kind of work are chiefly due to ill-fitting bands.

*Dr. Brown*—expressed pleasure at the small amount of criticism. The band of the crown is such that it can be trimmed to fit the root of any tooth. The flange on many roots should be removed before fitting the band.

*Dr. Shepard*—Thought that one statement of Dr. Brown's should be called in question : "that he had never set a crown with cement entire, and did not believe it could be successfully done"; he (Dr. Shepard) had set one hundred and fifty crowns, out of which number there were but two failures; in one case the crown broke, in the other the band was imperfectly fitted to the roots of a molar, and he simply used a cement to set the crowns. He believed that the failure in this class of work was owing largely to the miserable manner in which the neck of the root was fitted. Had recently removed several Richmond crowns, the bands of which were much too large to fit the roots.

*Dr. Williams*—Thought that Dr. Shepard stated a great truth when he said that a large per cent of the failurcs in crown work were due to poorly fitting bands. He has been engaged for a year past almost exclusively upon crown work, and so far as the Richmond process was concerned, when properly performed, the fit was perfect. Had seen two crowns removed from roots of teeth to attach some of better and more uniform color, in the case of Dr. Richmond, and the crowns had to be literally cut to pieces.

*Dr. Brown*—In reply to an inquiry concerning license to use the crown said that he would issue a license to any one man in a given locality who would stipulate and agree to not advertise it in any form or shape. He should not have his crown hawked into the public prints so long as he owned the patent, and he did not propose to sell it either.

The thanks of the society were unanimously voted Dr. Brown, for presenting his paper, charts, and models.

*Dr. Shepard*—Wished to exhibit some surgical instruments. Would first show a pair of guillotine forceps designed by Mr. Woodhouse, and manufactured by C. Ash & Sons. Mr. Woodhouse is the originator of this instrument, and not Dr. Frank Abbott of New York, as we are led to infer by a recent notice in the INDEPENDENT PRACTITIONER. The S. S White Dental Manufacturing Company have the patterns, and are to manufacture them. Also a pair of forceps for drawing forward the tongue during anesthesia, no wounding of the organ being possible; the instrument locking upon closure. Also a new form of syringe, clamp forceps, and clamp.

A paper was read by Dr. Geo. L. Parmele, of Hartford, Conn., "Concerning Records."

*Dr. Atkinson*—Wished to speak of Dr. Maxfield's paper upon Aconite. He thought it one of the finest papers and the best upon the subject he had ever heard. There was timidity and caution shown by the writer. The statement concerning Louis Jack, attributing to him the saying that aconite placed outside the gum will penetrate the tissues to the dental pulp, he thought was a mere assertion. It certainly should not be accepted until mentally digested.

Upon invitation, Dr. Shepard detailed his method of crowning teeth. The plate from which his bands are cut are usually $2.50 gold pieces rolled down to thirty-one guage. He used asbestos, ground fine as flour, with plaster, for investment of his work for soldering.

The next annual meeting will be its twenty-first, and it was voted to celebrate the occasion by an historical address, which Dr. Shepard was invited by the society to prepare and present. It was voted that a special invitation be extended to all "charter" members living, to be present at that meeting.

A communication from Dr. Jas. A. Bazin of Montreal, was read, relating to the meeting of the British Science Association at Montreal next August, and expressive of a desire for dentists and various dental societies to unite in that meeting. The communication was tabled for consideration at the June meeting.

Dr. C. T. Terry of Milan, Italy, was elected to honorary membership.

The officers elect were then installed.

It was voted to grant the president more time in the matter of appointing committees.

Adjourned subject to the call of the executive committee.

The following committees have since been appointed:

EXECUTIVE COMMITTEE.

Dr. Geo. A. Maxfeld, Holyoke, Mass.
Dr. F. W. Williams, Greenfield, Mass.
Dr. A. F. Davenport, No. Adams, Mass.

SECTION COMMITTEES.

1. Dr. E. S. Niles, Boston, Mass.
2. Dr. E. O. Wilbur, Thompsonville, Conn.
3. Dr. C. Fones, Bridgeport, Conn.
4. Dr. L. D. Shepard, Boston, Mass.
5. Dr. E. A. Stebbins, Shelburne Falls.

Reported by A. M. ROSS.

